# Short Occult Retractile Fibrous Scar Causing Exclusive Retrograde Stenosis of the Sigmoid Colon: An Incidental Diagnosis Nineteen Years After a Single Episode of Colonic Diverticulitis

**DOI:** 10.5334/jbr-btr.933

**Published:** 2016-02-15

**Authors:** Bruno Coulier, Raphael Rubay, Philippe Maldague, Isabelle Gielen

**Affiliations:** 1 Clinique Saint-Luc, Bouge, Belgium, BE; 2 Clinique St Luc, 5004 Bouge (Namur), BE; 3 Institute of Pathology and Genetics, Loverval, Belgium, BE

**Keywords:** Diverticulosis, Chronic diverticulitis, Colon stenosis, Abdomen, CT, Abdomen, ultrasound, Virtual colonoscopy

## Abstract

We report a rare case of purely retrograde stenosing stricture of the sigmoid descending colonic junction fortuitously diagnosed during the waning of a failed virtual colonoscopy in a 69-year-old patient. The rather asymptomatic patient was addressed to investigate a positive fecal occult blood test. He had suffered a single acute colonic diverticulitis episode 19 years before. A contrast-enhanced abdominal CT and complementary focused abdominal ultrasound fully diagnosed a short curvilinear contrast-enhancing “scar-like” tissue infiltrating the posterior colonic wall and developing retractile adherences with the retroperitoneum of the left iliac fossa. The imaging features are presented with pathologic correlation.

## Case Report

A 69-year-old man was referred to our Medical Imaging Department to undergo a virtual colonoscopy (VR). The patient had no specific abdominal complaint except mild chronic diarrhea worsened by metformin. He was referred to explain a positive fecal occult blood test. A conventional rectoscopy was normal, and a sigmoidoscopy was interrupted because of pain due to a spastic diverticular sigmoid. As he was taking clopidrogel (Plavix) for ischemic heart disease, there was a relative contraindication to perform a total optical colonoscopy (OC).

Almost immediately after starting the automated low-pressure colonic insufflation with carbon dioxide, the insufflator (with a maximal pressure set at 25 mm Hg) automatically stopped repeatedly, suggesting that there was a colonic stenosis or obstacle.

**Figure 1:**
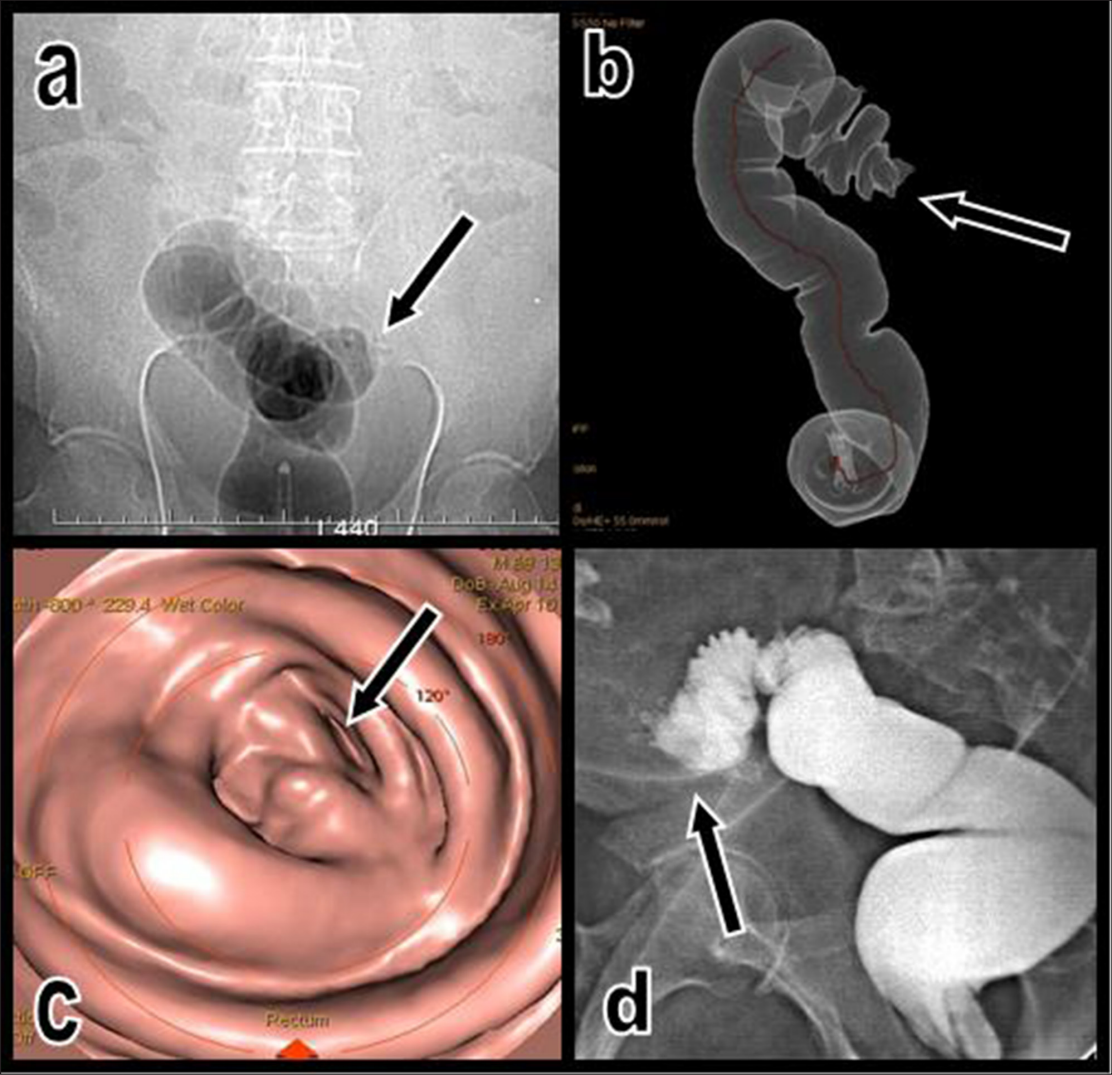
The topogram performed just after unsuccessful CO2 insufflation of the colon (a) illustrates an impassable colonic stenosis at the level of the junction between the sigmoid and descending colon (black arrow). A corresponding volume-rendering view (b) of the insufflated segment shows the abrupt cutoff (black arrow). Corresponding virtual endoscopic view of the cutoff (c). A classical colonic retrograde opacification obtained the next day after CT (d) also confirms the nearly complete cutoff of the colon proximal sigmoid; only a small amount of hydrosoluble contrast can pass through the stenosis (black arrow).

A complete cut off of the inflated colon was seen on a CT topogram at the level of the sigmoid and descending colonic junction (Figure 1). The virtual colonoscopy was immediately converted into a conventional non-contrast-enhanced abdominal CT and completed by secondary contrast-enhanced acquisition.

**Figure 2:**
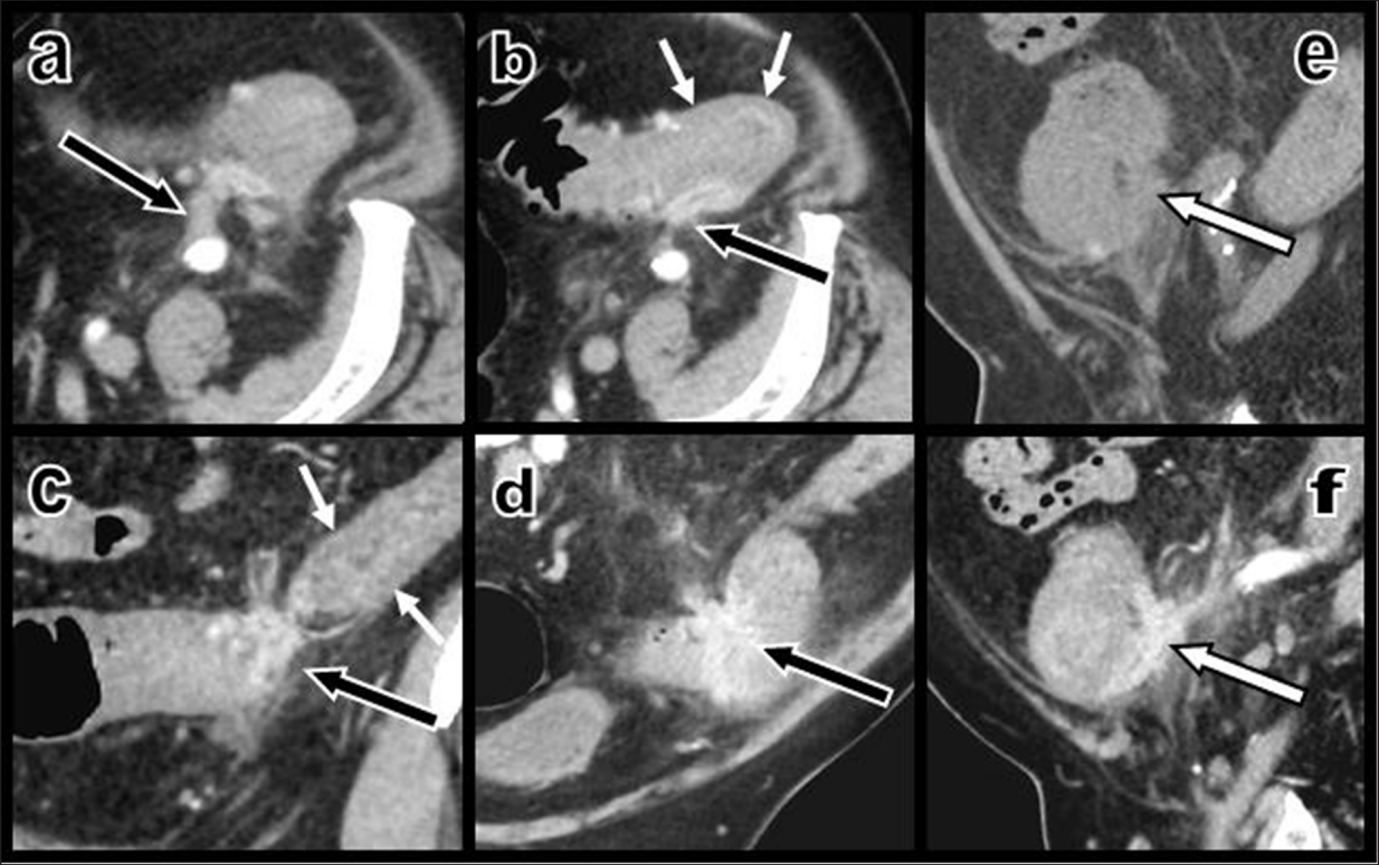
Contrast-enhanced axial CT views (a and b) and coronal oblique (c and d) multiplanar reconstructions (MPR) show a contrast-enhancing “scar-like” structure (black arrows) infiltrating the thickened colonic wall – due to a hypertrophy of the muscular layers (small white arrows) as confirmed by ultrasound – at the level of the sigmoid and descending colonic junction. This retracting tissue develops multiple adherences with the retroperitoneum. Sagital MPR views before (e) and after (f) intravenous iodine contrast injections illustrate the sharp enhancement of the curvilinear retractile “scar” (white arrow) that infiltrates the colonic wall and staples the colon on the retroperitoneum.

A short contrast-enhancing “scar-like” intra- and extraparietal structure was found infiltrating the posterior colonic wall at the level of the sigmoid and descending colonic junction (Figure 2). This curvilinear retractile “scar-like” tissue had developed multiple adherences with the retroperitoneum and the left iliac fossa and seemed to be stapling the colon on the retroperitoneum.

There was no obstruction, subocclusion, nor fecal stasis upstream, thereby suggesting a purely retrograde and unidirectional functional stenosis.

**Figure 3:**
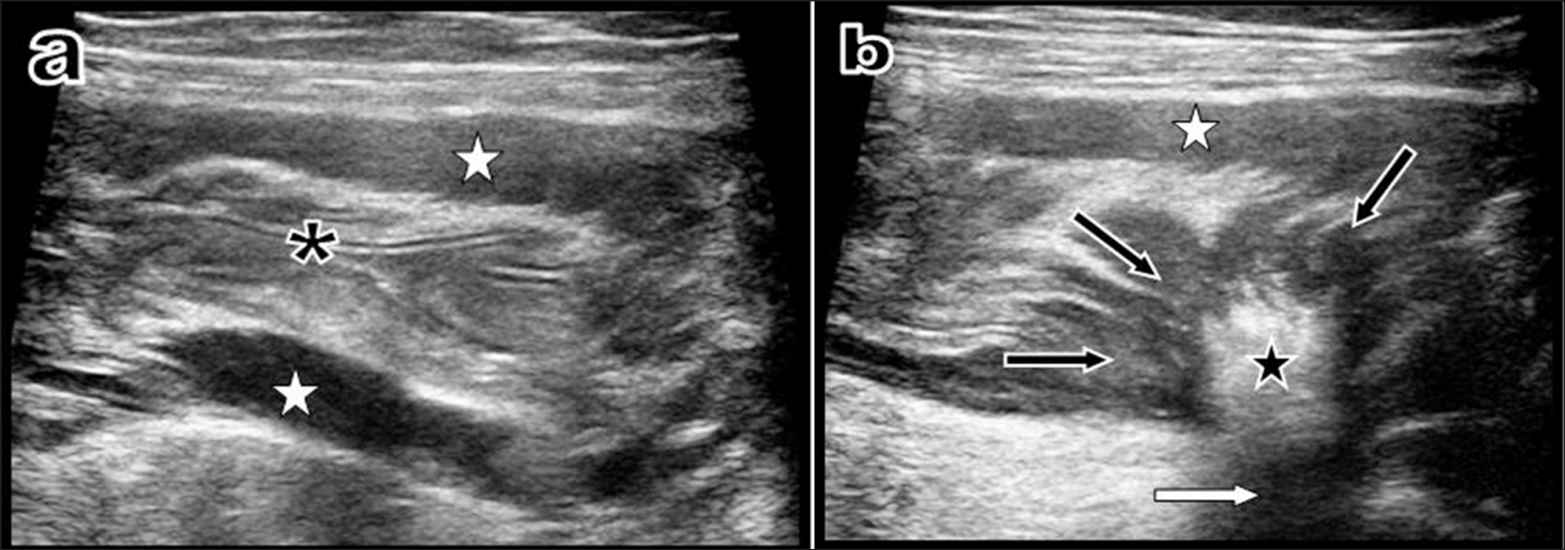
Longitudinal ultrasound view (a) of the descending colon just proximally to the stenosis shows that the colonic lumen is absolutely empty with normal mucosal folds (black asterisk). Nevertheless, hypertrophy of the muscular layers is evident (white stars). Another longitudinal ultrasound view (b) obtained just at the level of the retractile “scar-like” process shows the retractile centripedic convergence of the muscular layers (black arrows) towards a sharply well-delimitated hyperechoic (black star) and hyper-attenuating intraparietal scar (white arrow).

Additional ultrasound (Figure 3) study showed a perfectly empty descending colon with a normal mucosal relief, but with a thickening of the muscle layer. At the junction of the sigmoid and descending colon, ultrasound demonstrated a centripetal retractile convergence of the muscle layers to an intraparietal very hyperechoic and very attenuating scar.

The complete retrograde and unidirectional stenosis was confirmed by classical colonic opacification obtained the next day after CT. Only a small amount of hydrosoluble contrast could pass through the stenosis.

Retrospective anamnesis of the patient revealed a very old history of a single acute colonic diverticulitis episode 19 years before.

After a discussion with the multidisciplinary staff, a celioscopic resection of the stenotic segment was proposed to protect the patient from a prograde occlusion. The celioscopic resection was difficult due to retroperitoneal and left ureteral adherences, so a classical laparotomy became necessary. The postoperative period was uneventful.

**Figure 4:**
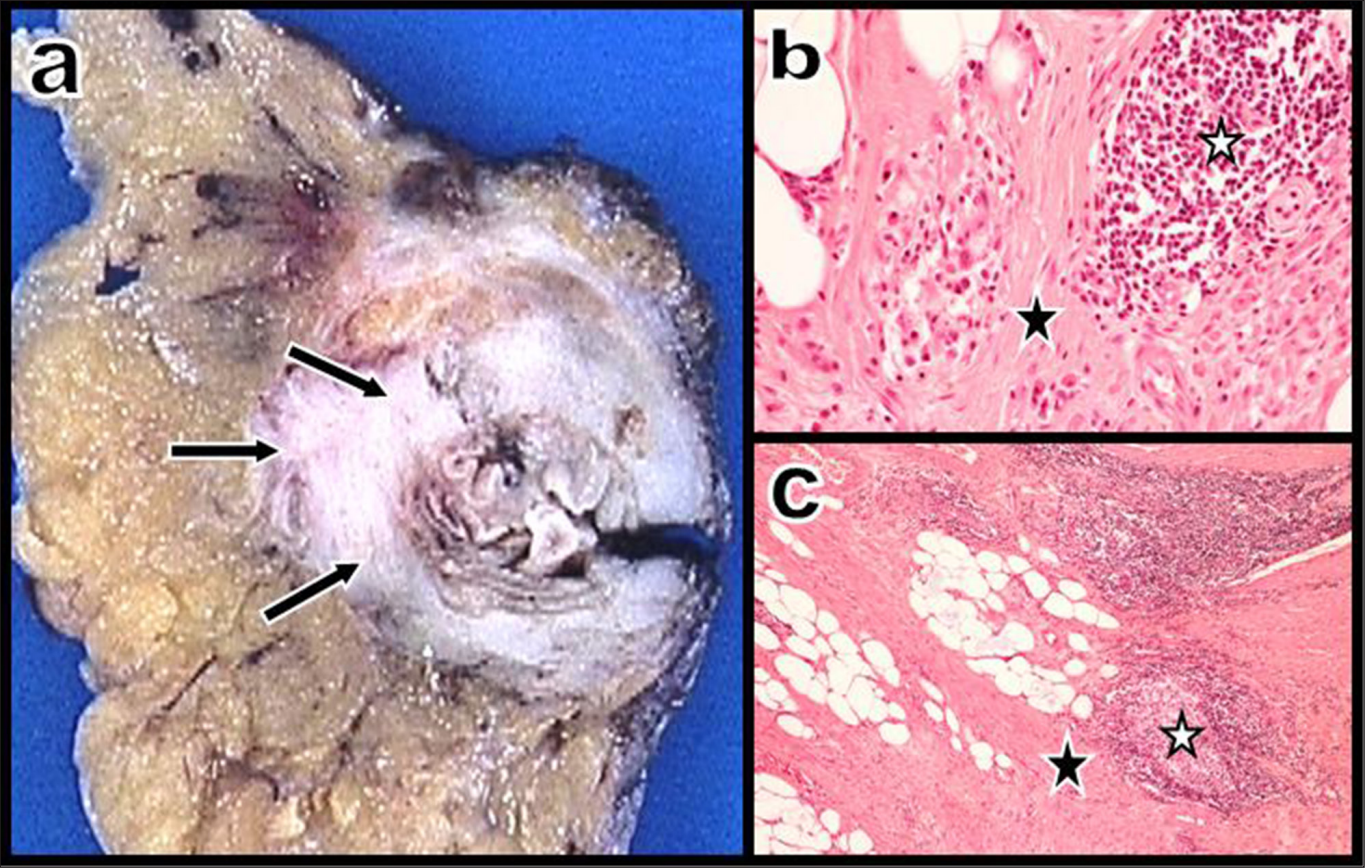
Gross anatomy (a) of the colonic wall shows a corbelling fibrosis infiltrating the wall of a diverticular segment. Photomicrographs – hematoxylin and eosin, × 20 (b) and × 5 (c) – show typical fibrosis (black star) with discrete chronic inflammatory infiltrates and lymphoid clusters (white stars).

Gross anatomy (Figure 4) of the resected colonic segment confirmed a short stricture constituted by corbelling fibrosis infiltrating the colonic wall of a diverticular segment. Histology showed typical fibrosis with discrete chronic inflammatory infiltrates and lymphoid clusters. Rare micro-abscesses related to chronic diverticulitis were also found.

Four months later, the patient was readdressed to the gastroenterologic staff to complete the study of the proximal colon which had not been studied. In the meantime, clopidrogel (Plavix) could be interrupted, and an optic colonoscopy was proposed to the patient. A small adenocarcinoma of the caecum was demonstrated – which was not visible on the CT – and a ceolioscopic right hemicolectomy was performed.

## Discussion

The incidence of diverticular disease of the colon has dramatically increased over the past century not only in the elderly but also among younger patients [1]. The disease is now a very common condition that affects nearly 50 per cent of adults over 60 years old in Western countries, and this prevalence increases to 70 per cent in the eighth decade [1,2,3].

It is estimated that about 10–25 per cent of people presenting colonic diverticulosis will develop one or more episodes of diverticulitis during their lifetime [1,2,4].

The clinical spectrum of diverticular disease is extremely variable, ranging from uncomplicated presentations, such as episodic pain or mild diverticulitis, to potentially life-threatening, complicated diseases, such as abscess, perforation, or hemorrhage. Episodes of mild nonspecific abdominal pain, often left-sided, can sometimes also be attributed to diverticular disease [1].

Most patients with classical acute diverticulitis have a lower quadrant attack with a constellation of clinical signs, including acute left lower quadrant pain, tenderness, fever, leukocytosis, and inflammatory syndrome, and abdominal CT is the most sensitive imaging technique for the diagnosis [2,4,5]. The most common classical complications of diverticulitis include abscess or phlegmon formation, fistula formation, stricture disease, bowel obstruction, and peritonitis.

Clinically, there is a more reduced subset of patients presenting only with chronic diverticulitis. The symptoms of these patients are typically much attenuated and far more indolent [2]. For the same reasons, these patients are more likely to first undergo a barium enema or optical colonoscopy rather than abdominal CT. These patients presenting with the chronic type of diverticulitis are more prone to develop chronic obstructive symptoms comprising bloating, constipation, decreased stool caliber, and nausea [2].

In our patient, virtual colonoscopy was preferred to optical colonoscopy because colonoscopy has an increased risk of perforation and bleeding in elderly patients and in those undergoing anti-coagulant therapy – as in our patient [5].

Virtual colonoscopy has the advantage of being technically feasible, well tolerated, and safe. Only patients with a positive finding will be referred for more invasive and risky examinations because of the high positive and negative predictive values of virtual colonoscopy for cancer and large polyps. Virtual colonoscopy also represents an alternative screening to evaluate patients with a positive fecal occult blood test who refuse optical colonoscopy [5].

In most patients with chronic diverticulitis, a barium enema reveals a relatively long segment of circumferential narrowing in the sigmoid colon with a spiculated contour and generally tapered margins. Sometimes an obstruction is present upstream of the stenosis. This situation again drastically differs in the reported case in which no symptoms of obstruction or fecal stasis were found and in which the stenosis appeared being very short, curvilinear, and asymmetrically retractile – instead of preferentially more long and circumferential in classical chronic diverticulitis [2]. Our patient had experienced only one episode of diverticulitis with acute symptoms about 19 years before and now had only very vague and occasional symptoms.

Colonic diverticulitis is supposed to be the cause of only 10 per cent of large bowel obstructions [5,6]. The large bowel can be obstructed in two ways: First, acute inflammation or edema of the pathologic segment eventually associated with pericolic abscess may narrow the lumen. Then, chronic inflammation, for example, after recurrent attacks of diverticulitis, can result in fibrous bands across the bowel lumen causing obstruction.

Such stricture or fibrous bands may be difficult to differentiate from obstructing neoplasm [6]. CT alone may not distinguish the benign from the malignant causes of luminal narrowing and colonoscopy. Sometimes surgery is required where diagnostic uncertainty remains.

Nevertheless, in the reported case, the curvilinear band-like aspect of the retractile stenosing process was very evocative of a fibrous band on CT views. Moreover, ultrasound was also very sensitive, excluding the presence of macroscopic tumoral mass and clearly showing a typical hyperechoic and hyperattenuating scar.

## Competing Interests

The authors declare that they have no competing interests.
